# Obstructive jaundice secondary to bile duct involvement with Hodgkin’s disease: a case report

**DOI:** 10.1590/S1516-31802005000100007

**Published:** 2005-01-02

**Authors:** Veruska Di Sena, Fernanda Prata Borges, Martins Thuler, Erika Pereira Macedo, Gustavo Andrade de Paulo, Ermelindo Della Libera, Angelo Paulo Ferrari

**Keywords:** Jaundice, Endoscopic retrograde cholangiopancreatography, Hodgkin’s disease, Lymphoma, Cholestasis, Icterícia, Pancreatocolangiografia retrógrada endoscópica, Doença de Hodgkin, Linfoma, Obstruções das vias biliares

## Abstract

**CONTEXT:**

Obstructive jaundice due to lymphoma is very rare. It may be difficult to distinguish between this condition and a large number of causes of extrahepatic bile duct obstruction, even by endoscopic retrograde cholangiography. Its prognosis is poor. Combined chemotherapy and/or radiotherapy with bile duct drainage is a therapeutic option.

**CASE REPORT:**

We describe a case of obstructive jaundice as the initial presentation of Hodgkin’s disease. After chemotherapy and endoscopic bile duct stenting, it was noted that the enlarged lymph nodes, jaundice and bile duct dilation disappeared.

## RESUMO

**CONTEXTO:**

Icterícia obstrutiva secundária a linfoma é muito rara. Em geral, a diferenciação com outras causas de obstrução biliar extra-hepática é difícil, mesmo com a colangiopancreatografia endoscópica retrógrada. Nestas condições o prognóstico é reservado. Associação de quimio, radioterapia e drenagem biliar é uma opção terapêutica a ser considerada.

**RELATO DE CASO:**

Descrevemos o caso de um paciente com icterícia obstrutiva como manifestação inicial da doença de Hodgkin. Após quimioterapia e drenagem biliar endoscópica, foi observado o desaparecimento da linfoadenomegalia, da icterícia e da dilatação da via biliar.

## INTRODUCTION

Jaundice concomitant with lymphoma may result from many causes.^1-4^ There are no recent references to the frequency of Hodgkin’s disease involving the bile ducts, but it is probably a rare event. One study published in 1980 reported a frequency of 0.5%.^1^ Non-Hodgkin’s involvement seems to be more frequent, with an incidence ranging from 0.5 to 2%. This unusual manifestation of lymphoma may easily be misdiagnosed.^1,3^ We present a case of obstructive jaundice as the first manifestation of Hodgkin’s disease, which resolved completely after endoscopic stenting combined with chemotherapy.

## CASE REPORT

A 46-year-old man was referred to our hospital for endoscopic retrograde cholangiopancreatography, with a nine-month history of epigastric pain, pruritus, jaundice, darkened urine and weight loss. On physical examination we noted that he was jaundiced, although in good general condition. His abdomen was soft and both the liver and spleen were enlarged.

His past medical history was notable with regard to non-Hodgkin’s lymphoma that had been diagnosed seven years earlier. After chemotherapy (CHOP regimen: cyclophosphamide, doxorubicin, vincristine and prednisone) and radiotherapy, he was considered cured. An abdominal computed tomography scan performed six months before the referral to our hospital demonstrated dilated intrahepatic ducts, hepatosplenomegaly, paraaortic lymphadenopathy and acalculous cholecystitis. Cholecystectomy and excisional paragastric lymph node biopsy had been done elsewhere and did not reveal any malignancy.

The initial laboratory tests upon admission to the hospital were: alkaline phosphatase 4,364 IU/l (normal < 250 IU/l); gamma glutamyl transpeptidase (GGT) 1,266 IU/l (normal < 32 IU/l); lactate dehydrogenase 529U/l (normal < 250 IU/l); aspartate aminotransferase (AST) 312 IU/l (normal < 35 IU/l); alanine amino-transferase (ALT) 309 IU/l (41 IU/l); and total bilirubin 11.4 mg/dl (normal < 0.4 mg/dl).

**Figure 1:**
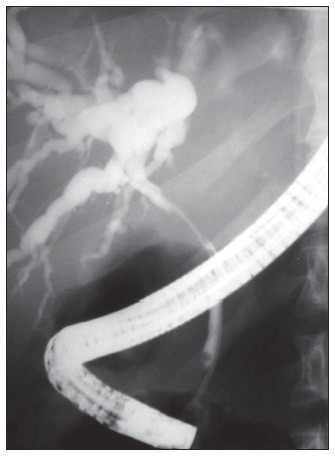
Initial endoscopic retrograde cholangiopancreatography showing a long narrow stricture involving the proximal common bile duct, with some intrahepatic dilatation, in an adult man with history of Hodgkin’s disease.

The endoscopic retrograde cholangiopancreatography revealed dilated intrahepatic ducts and an irregular area of narrowing, extending from the common hepatic duct to the proximal common bile duct (Figure 1). The diagnostic hypotheses at that time included cholangiocarcinoma, post-surgical or post-radiotherapy stricture and extrinsic compression. After contrast injection, a guidewire was placed through the stricture. Along the guidewire, cytology specimens were obtained using a Greene brush set. After brushing, a 10 Fr x 10 cm plastic stent was placed. Cytology brushing did not show malignant cells.

**Figure 2:**
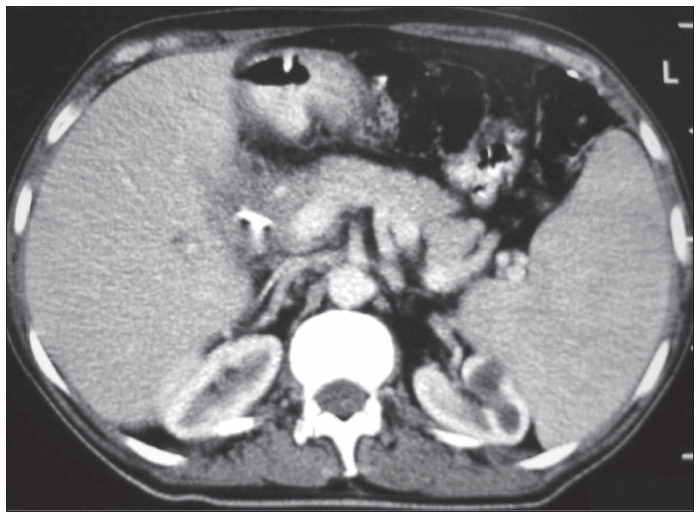
Computed tomography scan showing dilated intrahepatic ducts and splenomegaly, in a man with Hodgkin’s disease, four weeks after the placement of a plastic stent in a stricture of the bile duct.

**Figure 3:**
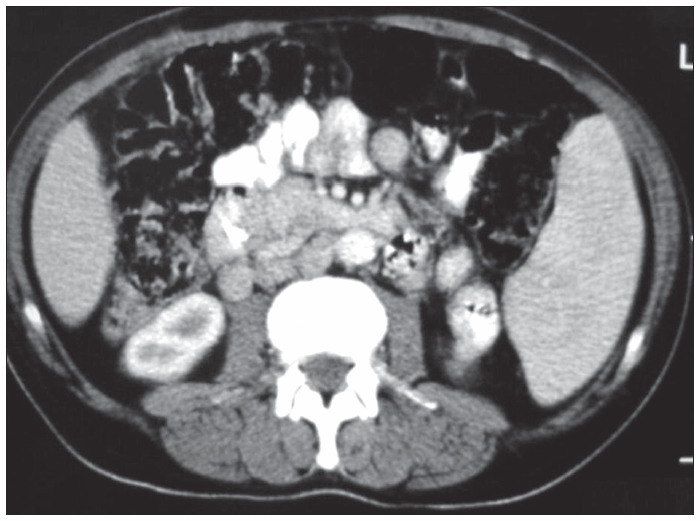
Computed tomography scan showing enlarged paraaortic lymph nodes, in a man with Hodgkin’s disease, four weeks after the placement of a plastic stent in a stricture of the bile duct.

After four weeks, a new abdominal computed tomography scan depicted dilated intrahepatic ducts, a soft mass surrounding the porta hepatis, splenomegaly and the same enlarged paraaortic lymph nodes (Figures 2 and 3). At this time, an enlarged inguinal lymph node was noted and excised. Histopathological examination revealed Hodgkin’s lymphoma of nodular sclerosis subtype, which stained positively for CD15 and CD30. Combination chemotherapy including doxorubicin, bleomycin, vincristine and dacarbazine (ABVD regimen) was begun.

**Figure 4:**
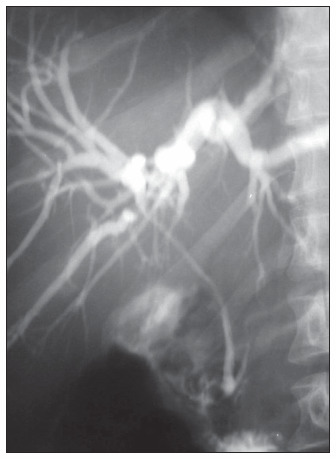
Endoscopic retrograde cholangiopancreatography in an adult man with history of Hodgkin’s disease after chemotherapy showing complete resolution of the bile duct stricture, with no intrahepatic dilatation.

One month later, he was asymptomatic and his endoscopic retrograde cholangiopancreatography was normal (Figure 4). He has now been off the therapy for 13 months, and has been doing well for the last 18 months.

## DISCUSSION

Jaundice concomitant with lymphoma may result from many causes. Hepatitis, hemolysis, common bile duct stones, lymphomatous involvement of the liver and an unusual syndrome of cholestasis without liver or bile duct involvement have all been described.^1-4^

Extrahepatic bile duct obstruction by lymphoma is a rare cause of jaundice. In one series of 370 patients, evidence of lymphomatous obstruction was present in only five of them (1.3%).^1^ This may occur either as an initial or a late manifestation of the disease.^3^

Bile duct involvement may occur in various forms. Primary malignant non-Hodgkin’s lymphoma of the common bile duct and also primary infiltration of this by Hodgkin’s disease have been reported. Primary lymphoma arising in the porta hepatis has also been described.^5^ However, most frequently, lymphomatous nodes extrinsically compress the extrahepatic bile duct.^1-4^

Abdominal ultrasonography and computed tomography scanning are both excellent techniques for bringing bile duct dilatation into view. However, they can fail to detect localized duct dilatation.^3^

Endoscopic retrograde cholangiopancreatography and percutaneous transhepatic cholangiography are more sensitive for detecting minimal or focal duct dilatation, thereby allowing cytology brushing and/or stent placement.

Regardless of whether the obstruction develops primarily or secondarily, the diagnosis of extrahepatic bile duct obstruction related to lymphoma remains difficult.^1^ The appearance of these lesions can simulate common duct stones, pancreatic cancer, sclerosing cholangitis, cholangiocarcinoma and benign strictures following radiotherapy, as in our patient.^1,3^ Stenotic bile duct disease secondary to *Cryptosporidium* or cytomegalovirus has been described in patients with acquired immunodeficiency syndrome (Aids) and is a potential complication in immunosuppressed patients with lymphoma.^3^

The case reported illustrates a diagnostic dilemma, because the patient developed obstructive jaundice seven years after the original diagnosis of lymphoma had been made. Moreover, the endoscopic retrograde cholangiopancreatography images were not characteristic of intrinsic or extrinsic compression, and therefore cholangiocarcinoma and post-radiotherapy stricture were first considered. There is no certainty that the common bile duct itself was affected by the lymphoma. Negative cytology brushing does not exclude such a possibility.

The majority of Hodgkin’s or non-Hodgkin’s lymphomas show moderate to marked regression early in the course of chemotherapy and/or radiotherapy, and obstructive jaundice may resolve without drainage.^1^ However, bile duct drainage allows the use of full dose chemotherapy. Complications related to stenting may cause delays in the primary anticancer treatment.

There are few reports of stent placement in obstructive jaundice caused by lymphoma, and most of such stenting was inefficient.^1^ One point to consider is that many agents used for treating lymphomas are hepatotoxic and hence contraindicated in patients with jaundice. The combination of percutaneous transhepatic bile duct drainage with radiation or chemotherapy has proven to be extremely effective in some cases.^3,4^ In our patient, the cholestasis was resolved after endoscopic stent placement and full doses of ABVD could be administrated. It is worth remembering that endoscopic bile duct stenting has lower complication rates than for percutaneous stenting.

An association between Hodgkin’s and non-Hodgkin’s lymphoma, as in our patient, has been described as the occurrence of either sequential events or at the same site, in which case they are referred to as *composite*.^6,7^ The most common association is between lymphocyte predominance and large-cell lymphoma, but associations of other subtypes have also been reported.^8^

Primary or secondary bile duct obstruction is considered a poor prognostic sign for non-Hodgkin’s lymphoma.^1,3^ Otherwise, Hodgkin’s disease seems to have a better prognosis, as in the present case.^1^

We have presented a case report that reminds us that the diagnosis of Hodgkin’s disease should be added to the large list of possible etiologies in malignant bile duct obstruction.
